# Geographical variations of cancer incidence in Guadeloupe, French West Indies

**DOI:** 10.1186/s12885-022-09886-6

**Published:** 2022-07-18

**Authors:** Bernard Bhakkan-Mambir, Jacqueline Deloumeaux, Danièle Luce

**Affiliations:** 1 Registre général des cancers de Guadeloupe, Centre Hospitalier Universitaire de la Guadeloupe, Guadeloupe F.W. I. Route de Chauvel, 97159 Pointe-à-Pitre Cedex, France; 2 INSERM, EHESP, Université de Rennes, IRSET (Institut de recherche en santé, environnement et travail)- UMR_S 1085, F-97100 Pointe à Pitre, France

**Keywords:** Spatial analysis, cancer, incidence, Caribbean

## Abstract

**Background:**

Geographical disparities in cancer incidence are observed at different scales and may highlight areas of high risk that need special attention to improve health policies. In Guadeloupe, a French archipelago in the Caribbean, environmental and socioeconomic factors are potential factors associated with cancer incidence. Our objective was to describe geographical variations of cancer incidence in Guadeloupe at a small-area level, in order to identify potential clusters.

**Methods:**

We conducted spatial analyses for the 18 most frequent cancer sites, using data collected by the population-based cancer registry of Guadeloupe over the period 2008–2017. For each cancer sites, we used the Besag, York and Mollié model to estimate smoothed standardized incidence ratios (SIRs) at a sub-municipality level. In addition, we performed ascendant hierarchical clustering of these smoothed SIRs to describe the relationship between the different cancer sites and to identify geographical clusters.

**Results:**

We observed geographical disparities with a spatial pattern that varied across cancer sites. Clustering of the smoothed SIRs showed aggregations between breast cancer and multiple myeloma, thyroid and stomach cancer, cervical and head and neck cancers, lung and rectal cancers, ovarian and endometrial cancers. Cluster analysis also identified six geographical clusters. Features of these clusters suggest alcohol consumption, exposure to pesticides, pollution generated by open landfills, and ethnicity as possible explanatory factors.

**Discussion/conclusion:**

Our study provided for the first time an extensive description of geographical disparities in cancer incidence in Guadeloupe, in a region where socioeconomic and environmental issues are major concerns. Although the identification of underlying factors was out of the scope of the present study, we highlighted areas of special interest and put forward some hypotheses that warrant to be further investigated in more in-depth analyses.

**Supplementary Information:**

The online version contains supplementary material available at 10.1186/s12885-022-09886-6.

## Background

With more than 19 million new cases and nearly 10 million deaths in 2020 [1], cancer is a major cause of morbidity and mortality in the world. Cancer is a multifactorial disease and causes include genetic, infectious, lifestyle and environmental risk factors. Geographical variation of cancer incidence is observed at different scales. Spatial analysis at a small-area level may highlight areas of high risk that need special attention and may improve health policies such as resource planning or access to preventive care. Geographic patterns for each cancer site may also provide clues for possible risk factors and help formulate etiological hypotheses. These hypotheses can be refined if several aggregates show similarities, and the analysis can be supplemented by analyzing several cancers simultaneously. In particular, cluster analysis, although rarely used, is an exploratory tool to classify the geographical areas according to the correlation between the incidence of different cancer sites [2], which could allow identifying shared risk factors.

Guadeloupe is a French overseas territory in the Caribbean, composed of five islands (Grande-Terre, Basse-Terre, Les Saintes, Marie-Galante, La Désirade) with a population of about 400,000 inhabitants (Fig. S1). The population is multi-ethnic but mostly from African descent. The population faces a high prevalence of cardiovascular and metabolic diseases, with 48% of the population being overweight or obese [3]. Cancer incidence is generally lower than in metropolitan France, but it is higher for prostate, stomach and cervical cancers. In general, the incidence rates of cancers in Guadeloupe are between those of metropolitan France and those of the other Caribbean countries [1]. This is reflected in the prevalence of cancer risk factors, which is in the French West Indies in-between mainland France and other Caribbean territories [4] In Guadeloupe, the prevalence of daily tobacco smoking is 12%, the prevalence of daily alcohol drinking is 6.3% [3]. Socioeconomically, compared to the French national average, the population of Guadeloupe has a lower median income, a lower educational level, a higher rate of unemployment, and a larger proportion of people who get income support [5]. The French health insurance system provides in principle universal social security coverage. From an environmental point of view, Guadeloupe suffers from natural phenomena such as sand mists from the Sahara [6] or the invasion of brown sargassum algae [7] and is characterized by an extensive use of pesticides [8].

Our objective was to describe geographical variations of cancer incidence in Guadeloupe, at a small-area level in order to identify potential clusters.

## Material and methods

### Data

We used data from the Guadeloupe cancer registry, which collects all new cancer cases of patients residing in Guadeloupe since 2008. The registry routinely collects for all cancer sites: the date of diagnosis, the topographical and morphological codes of the International Classification of Diseases for Oncology, (ICD-O3) and sociodemographic data (sex, date of birth and exact address of the place of residence). The registry belongs to the French Network of Cancer Registries (FRANCIM) and meets high quality criteria; the completeness and data quality are regularly assessed by the “Comité d'évaluation des registres” (CER).

We conducted spatial analysis for the 18 most frequent cancer sites: prostate (code ICD-O3 C61; 4418 cases), breast (C50; 1772 cases), colon (C18-C19;1007 cases), rectum (C20_C21;272 cases), stomach (C16;712 cases), esophagus (C25;178 cases), pancreas (C15;246 cases), liver (C22;141 cases), multiple myeloma and plasmocytoma (C42.1 and morphology codes 9731 to 9734; 669 cases), cervix (C53;218 cases), uterus corpus (C54;334 cases), ovary (C56;148 cases), kidney (C64;190 cases), brain (C71;102 cases), thyroid (C73;190 cases)), upper aero-digestive tract (UADT) (C00 to C14 and C30 to C32;527 cases) and melanoma (C44 and morphological code from 8720 to 8770;91 cases). We studied only invasive tumors over the period 2008–2016, i.e., 12,397 tumors.

As a geographical level, we used a sub-municipality level, the IRIS level (for Ilots regroupés pour l’information statistique; Merged Islet for Statistical Information). The IRIS is the smallest geographical census unit available in France. There are 136 IRIS in Guadeloupe. For each patient, the address was geocoded to assign the IRIS of residence. Population for each IRIS by sex and age group (0–14; 15–29; 30–44; 45–59; 60–74; ≥75) was obtained from census data for the years 2008–2016.

### Statistical analysis

We first conducted spatial analyses of cancer incidence for the 18 main cancer sites. We used the Besag, York and Mollié model [9] to estimate smoothed SIRs (Standardized Incidence Ratios) for each IRIS. Let O_i_ the number of observed cases in IRIS i, assumed to follow a Poisson distribution, E_i_ the number of expected cases calculated with indirect standardization for age and sex, θ_i_ = O_i_/E_i_ the SIR in IRIS i. The BYM model is written as follows:

log $${\hat{\theta}}_i$$ = *α* + *u*_*i*_ + *v*_*i*._Where:

*α* is the intercept

*u*_*i*_ represents the non-spatial random effects and ${u}_i\sim N\left(0,{\sigma}_u^2\right)$*, v*_*i*_ represents the spatial heterogeneity and was modeled by a conditional autoregressive model (CAR).

The convergence of the model was verified with the Geweke criterion [10]. The graphical representation and the BYM model were implemented with WINBUGS.1.41 and R.4.0.2.

We then performed ascendant hierarchical clustering (AHC) of the smoothed SIRs, using Ward’s method implementation (ward. D2) in the hclust function of R stats package [11]. Two AHCs were performed [2]. First, in order to highlight the relationships between the incidence of different cancers, we performed an AHC of the 18 cancer sites. We also calculated Spearman’s correlation coefficients between the smoothed SIRs. Then, we performed an AHC of the 136 IRIS in order to define geographical clusters. The optimal number of clusters was determined with the elbow method using total within-cluster sum of square as criterion and from examination of the clustering dendrogram.

## Results

Table 1 shows for all cancers and several cancer sites the estimated age-standardized incidence rates in 2020 for Guadeloupe, mainland France, the United States and the Caribbean [1]. Guadeloupe has the highest standardized incidences for prostate and stomach cancers, and the lowest for cancers of the corpus uterus, head and neck, liver, lung, and brain.

**Table 1:** Age-standardized incidence rates (World) per 100,000 in 2020, both sexes

	Guadeloupe	USA	Caribbean	France
Breast	67.9	90.3	50.9	99.1
Cervix Uteri	7.9	6.2	13.7	7.0
Corpus Uteri	9.7	21.4	12.7	14.9
Ovary	8.3	8.1	4.6	6.9
Prostate	183.6	72.0	75.8	99.0
Stomach	10.7	7.9	6.9	4.7
Liver	4.0	6.9	5.5	7.6
Colon-rectum	21.9	25.6	18.2	30.1
Esophagus	2.9	2.8	2.8	3.6
Pancreas	8.3	8.2	4.3	8.6
Lung	11.3	33.1	17.6	34.9
Brain	2.7	5.4	3.3	6.7
Thyroid	4.5	11.8	3.9	14.8
Head and neck	8.6	10.3	10.0	16.3
Multiple myeloma	5.0	4.9	2.3	4.2
Melanoma	1.5	16.6	0.7	15.2
Kidney	2.5	12.4	2.7	11.2
All cancers	258.6	362.2	191.7	341.9

Data were extracted from the Globocan 2020 database [1]

### Smoothed SIRs by cancer site

**Fig. 1:**
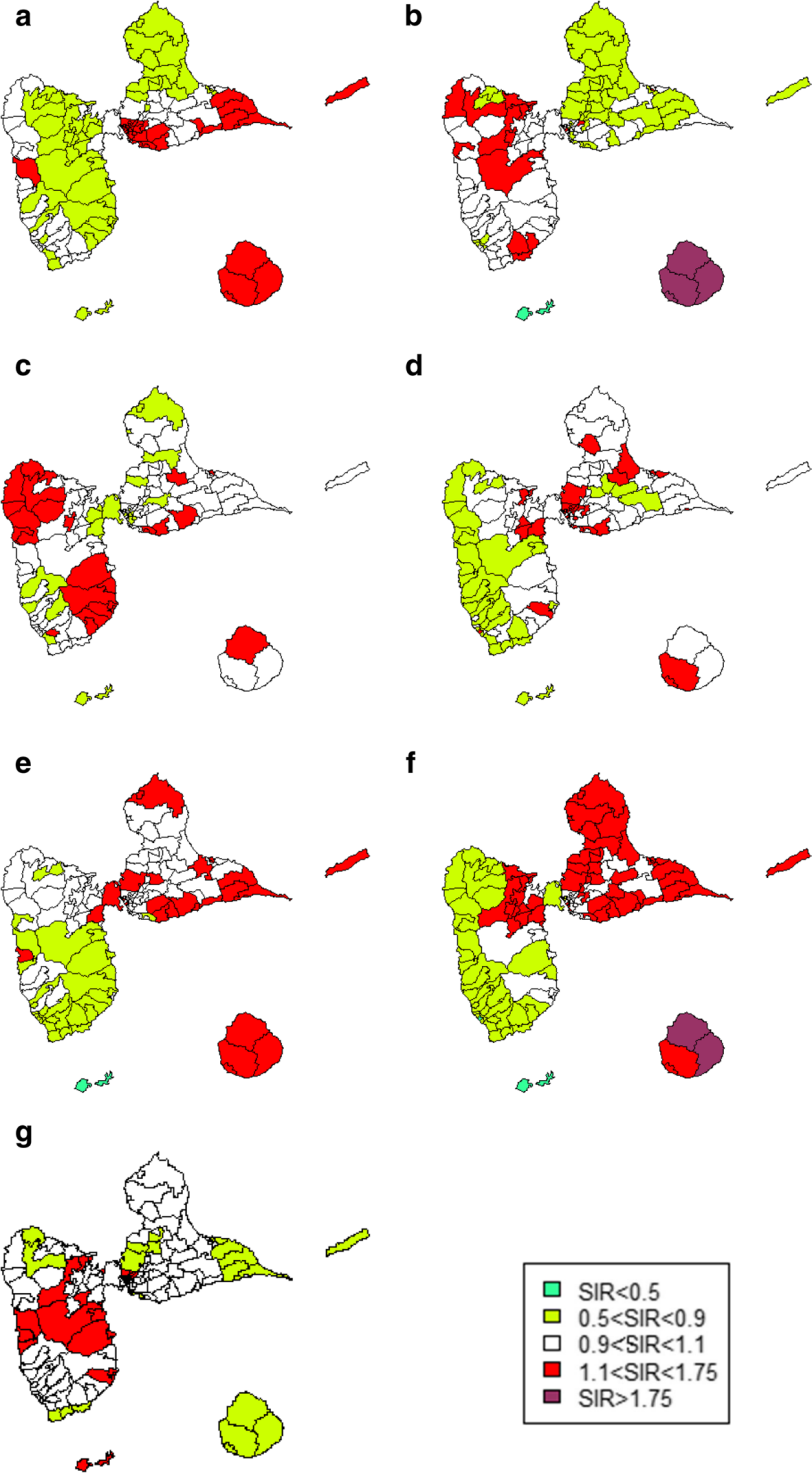
Smoothed SIRs for head and neck and digestive cancers. **a** Head and neck cancer. **b** Esophageal cancer. **c**- Stomach cancer. **d** Colon cancer. **e** Rectal cancer. **f** Liver cancer. **g** Pancreatic cancer

Excess risks of head and neck, colon, rectum and liver cancers were observed in the eastern part of Guadeloupe (Grande-Terre Island). The western part of Guadeloupe (Basse-Terre) showed an excess risk of esophageal, stomach and pancreatic cancers. Marie-Galante, the island located in the south-east had high incidence areas for all digestive cancers, excluding those of the pancreas, as well as for head and neck cancers. In this territory SIRs were particularly elevated for esophageal and liver cancers (Fig. 1).

**Fig. 2:**
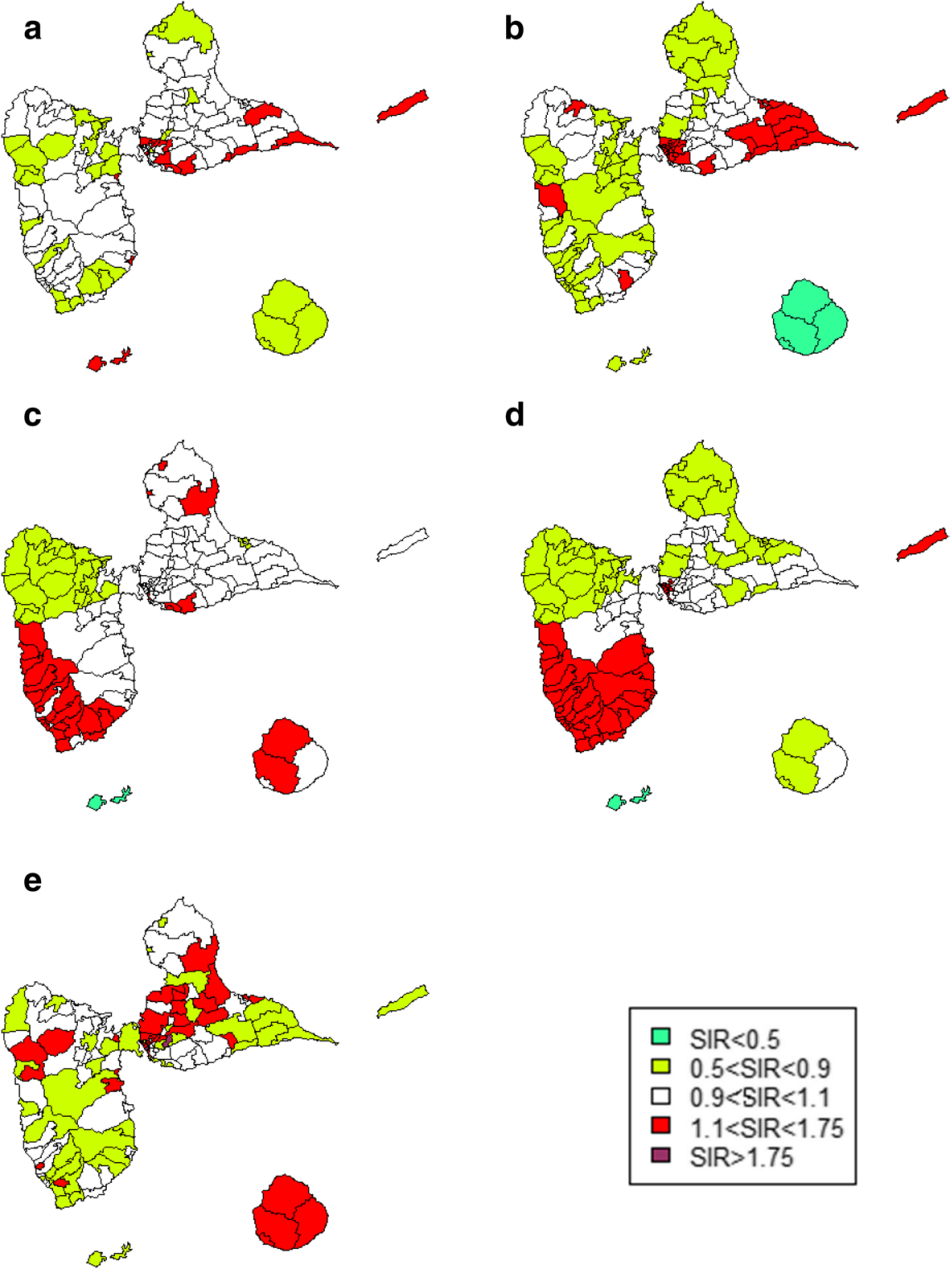
Smoothed SIRs for urogenital cancers. **a** Breast cancer. **b** Cervical cancer. **c** Uterine corpus cancer. **d** Ovary cancer. e Prostate cancer

High incidence areas for cancers of the corpus uteri and ovary were mainly found in Basse-Terre. The areas of high incidence of breast and cervical cancers were mostly found in Grande-Terre. Areas with an excess risk of prostate cancer were in the center of Grande-Terre, in the north of Basse Terre and in Marie-Galante. La Désirade, the island located to the east, presented an excess risk of breast, cervical and ovarian cancers (Fig. 2).

**Fig. 3:**
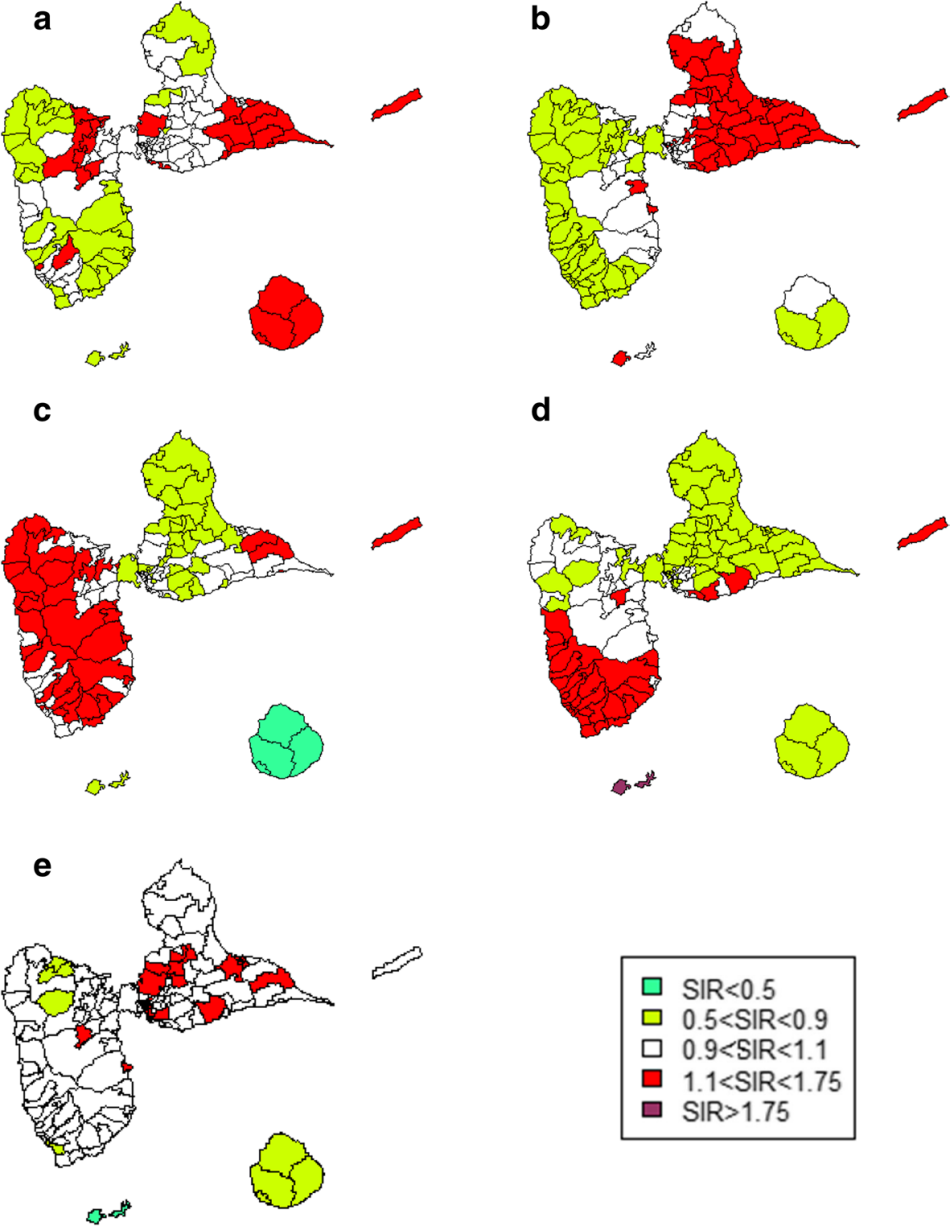
Smoothed SIRs for other cancers. **a** Lung cancer. **b** Brain cancer. **c** Thyroid cancer. **d** Melanoma. **e** Multiple myeloma

Regarding other cancer sites, areas with an excess risk of brain cancer and multiple myeloma were mainly found in Grande-Terre, whereas Basse-Terre had the majority of the areas with an excess risk of thyroid cancer and melanoma. La Désirade was an area of excess incidence for lung cancer, melanoma, brain cancer and thyroid cancer. (Fig. 3)

### Cluster analysis

**Fig. 4:**
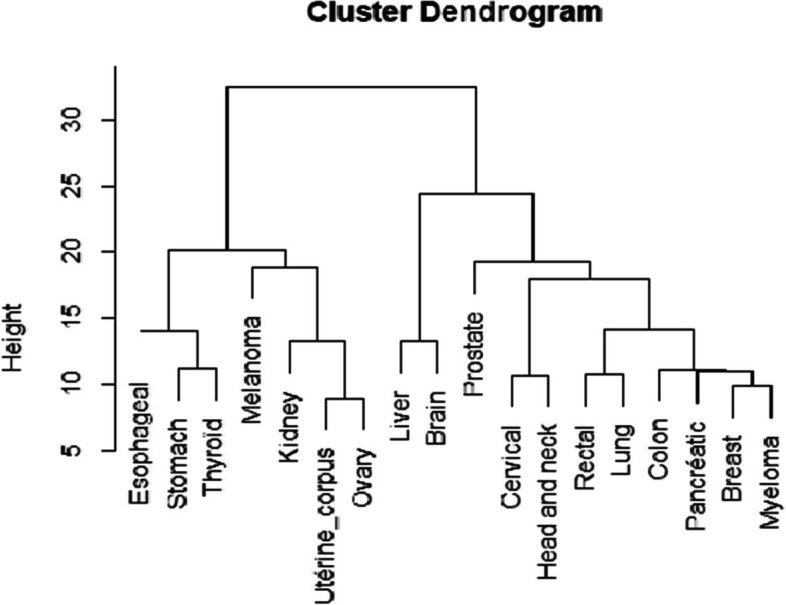
Dendrogram showing the relationships between the smoothed SIRs of the 18 cancer sites

**Fig. 5:**
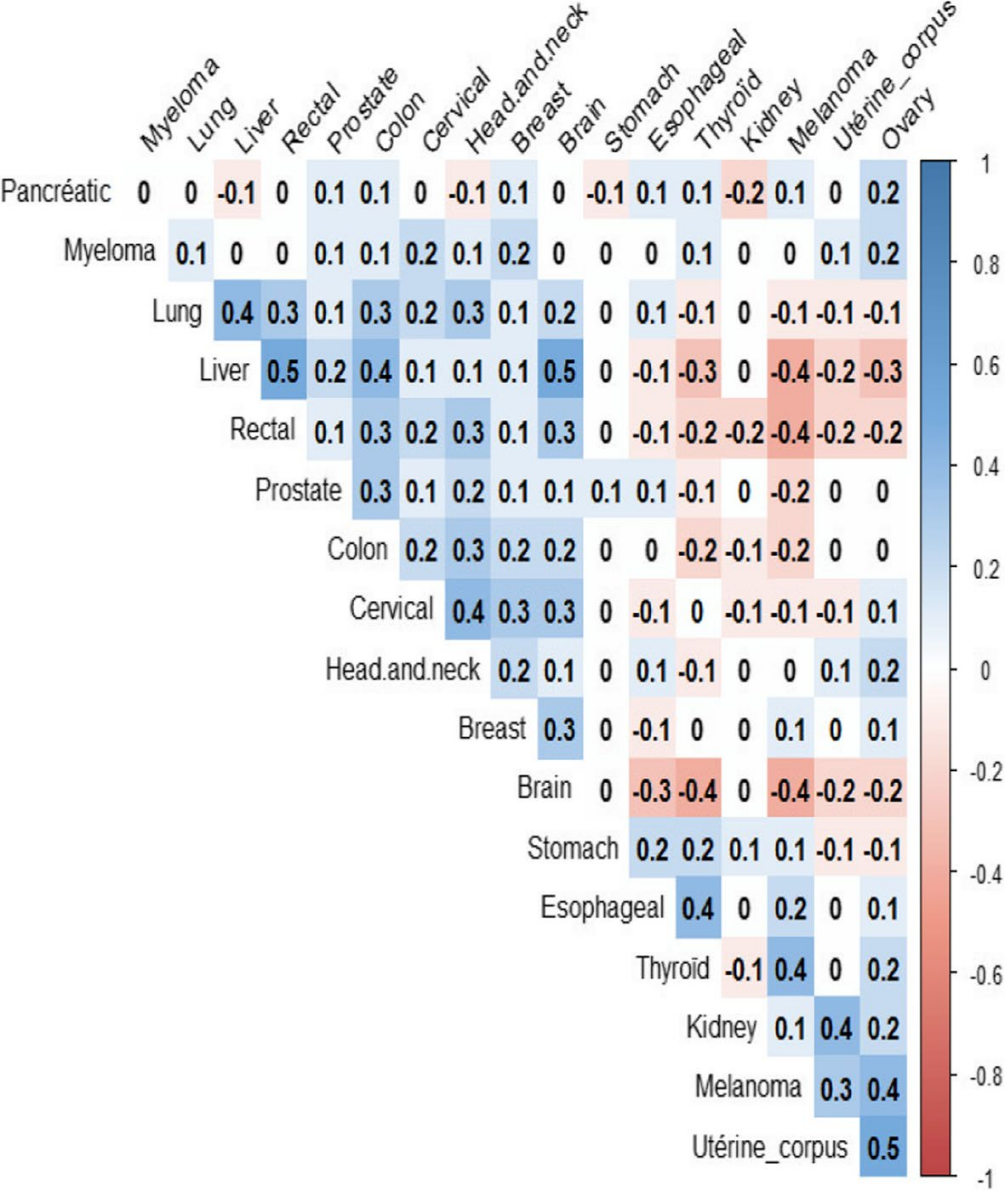
Spearman’s correlation coefficients between the smoothed SIRs

Figure 4 represents the dendrogram of the ascending hierarchical clustering of the 18 cancer sites. The more marked aggregations were found between breast cancer and myeloma, thyroid and stomach cancer, cervical and head and neck cancers, lung and rectal cancers and ovarian and corpus uteri cancers. Associations between these cancer sites are supported by the correlation coefficients between the smoothed SIRs (Fig. 5).

**Fig. 6:**
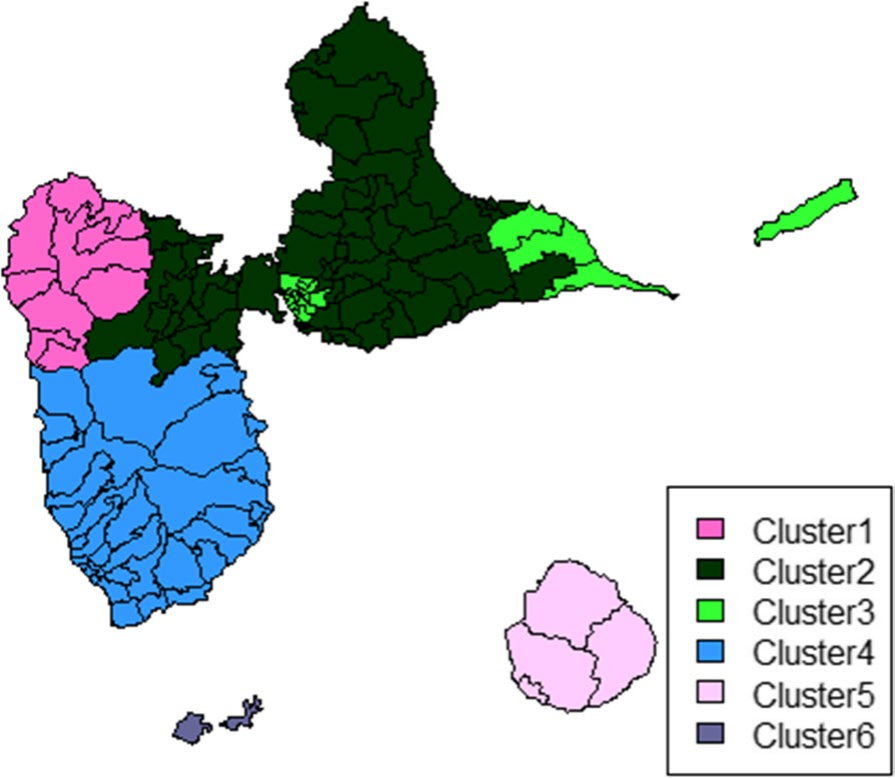
Clustering of the IRIS based on smoothed SIRs of different cancers

The ascending hierarchical clustering of the 136 IRIS resulted in an optimal number of 6 classes. Figure 6 shows the geographical representation of these 6 clusters. In order to describe the profile of each cluster we calculated the average smoothed SIRs by cancer site (Table 2). Cluster 1 was characterized by a high incidence of stomach and thyroid cancers and a low incidence of cancers of the uterus, ovary, liver, brain and kidney. Cluster 2 presented high SIRs for liver and brain cancers. In Cluster 3, SIRs were above 1 for most cancer sites, and were particularly high for cervical and head and neck cancer. Cluster 4 showed a high incidence of cancer of the uterus, ovary, thyroid, melanoma and kidney. Cluster 5 presented high SIRs for most digestive cancers and head and cancer, as well as for lung, brain and kidney cancer. On the other hand, the incidence of breast and cervical cancer, multiple myeloma and melanoma was low. Cluster 6 was characterized by a low incidence of most cancer sites, and a very high incidence of melanoma, and to lesser extent of breast, pancreatic and brain cancers. We also examined sociodemographic characteristics of the 6 clusters (Table S1). The proportion of women was the highest in cluster 3 and the lowest in cluster 6. The population was older in clusters 5 and 6 than in the other clusters. The proportion of unemployed varied from 15.2% in cluster 6 to 23.6% in cluster 1. Educational level was lower in clusters 5 and 6. These two clusters were also characterized by a higher proportion of farmers and fishermen.

**Table 2:** Average smoothed SIRs by cancer site for each cluster

	Cluster 1	Cluster 2	Cluster 3	Cluster 4	Cluster 5	Cluster 6
Breast	0.95	1.00	1.11	0.97	0.66	1.30
Cervix uteri	0.97	0.98	1.36	0.91	0.29	0.68
Corpus uteri	0.70	0.97	1.02	1.12	1.12	0.41
Ovary	0.67	0.92	1.13	1.22	0.88	0.00
Prostate	0.96	1.05	1.08	0.93	1.26	0.71
Stomach	1.20	0.98	0.93	1.02	1.02	0.75
Liver	0.60	1.17	1.08	0.66	1.76	0.00
Colon	0.89	1.04	1.15	0.87	1.20	0.63
Esophagus	1.07	0.91	0.94	1.01	2.53	0.00
Rectum	0.98	1.04	1.10	0.84	1.30	0.41
Pancreas	0.93	0.98	1.05	1.02	0.85	1.17
Lung	0.85	1.03	1.09	0.90	1.22	0.66
Brain	0.71	1.13	1.13	0.76	0.86	1.16
Thyroid	1.27	0.95	0.99	1.12	0.31	0.68
Head and neck	0.91	0.95	1.25	0.91	1.63	0.64
Multiple myeloma	0.92	1.04	1.13	0.98	0.54	0.34
Melanoma	0.92	0.83	0.90	1.29	0.71	4.48
Kidney	0.74	1.00	0.90	1.15	1.14	0.06

## Discussion

We observed geographical disparities, with a spatial pattern that varied across cancer sites. Environmental, socioeconomic factors, as well as ethnicity may partially explain these disparities. Our objective was not to identify precisely the underlying causes, but rather to provide leads for further study and to generate hypotheses. Nevertheless, some potential explanatory factors are briefly discussed below.

For prostate cancer, the most frequent cancer in Guadeloupe, no clear spatial pattern emerged. Prostate cancer has been related to exposure to pesticides in general, and in Guadeloupe particularly has been found to be associated with chlordecone, an insecticide previously used in banana plantations [12]. However, the spatial distribution of prostate cancer cases does not specifically match agricultural areas, located in the South East of Basse Terre (banana) and the North of Grande Terre (sugarcane). Instead, high incidence areas were found in urban areas including most health care facilities, which may suggest a role of opportunistic screening. For breast cancer, the second cancer in Guadeloupe, with an incidence rate however lower than in European countries, most SIRs were below 1.1, except for a limited number of high incidence areas, which are characterized by a high proportion of population of European origin, and/or the presence of open landfills.

For digestive cancers, the spatial distribution of cancers of the colon differed markedly from that of rectal cancers. The cancer sites are often grouped in epidemiological studies, despite possible differences in risk factors [13]. The incidence of stomach cancer is high in Guadeloupe, and within Guadeloupe the highest SIRs were found in the North-West and South-East of Basse-Terre. These areas comprise most of the banana plantations and are also characterized by a high social deprivation [14] possibly related to a high prevalence of *helicobacter pylori*.

Besides the spatial analysis of each cancer site, results of the cluster analysis may help formulate hypotheses by identifying areas with high incidence of several cancer sites that may share common risk factors. Some of the six identified clusters warrant further attention. Cluster 1, in the North of Basse-Terre, is characterized by a high incidence of stomach and thyroid cancer, for which we were not able to identify a common risk factor. Cluster 2 in the center of Guadeloupe, is an area where the majority of sugarcane plantations are located as well as the main sugar and rum factories. This area showed an excess incidence of liver cancer, the main risk factor being alcohol consumption [15], and an excess incidence of brain cancer, potentially related to exposure to pesticides [16]. Cluster 3 is the only cluster composed of two non-adjacent parts. A common feature of these two parts is a high population density. Living in an urban area is a risk factor for breast and cervical cancers [17]. The sedentary lifestyle associated with urban living could explain the higher incidence of colon cancer [18]. Another potential risk factor could be the pollution generated by the open landfills present in the two distinct areas. Finally, the high incidence of head and neck and cervical cancer observed in this cluster may be due to a high circulation of Human Papilloma Virus (HPV), associated with both cancer sites [19, 20]. Cluster 4 includes large areas with banana plantations, with an extensive use of pesticides, including endocrine disruptors such as the organochlorine insecticide chlordecone. Several cancers (ovarian, endometrial, and thyroid) displaying a high incidence in this area are hormone-sensitive [21]. However, there was no excess of breast and prostate cancer. This may be partially explained by the relatively large proportion of people from Indian descent in the cluster population, for whom reported incidence rates of breast and prostate cancer are usually lower than for Afro-descendent people.

Cluster 5 corresponds to the island of Marie-Galante, with many sugar cane plantations and a sugar factory. This area showed the highest incidence for most of the cancer sites, particularly for alcohol-related cancers [15]. Unfortunately, data on alcohol consumption in Guadeloupe are only available for the whole population, and are not collected at any geographical level. Cluster 6 corresponds to the islands of Les Saintes, where the population is from European descent in its vast majority. This may explain the higher incidence of breast cancer [22] and melanoma [23]. In addition, sun exposure in fishermen, an important activity in these islands, may also contribute to the high incidence of melanoma.

An important limitation of our study is the relatively small number of cases for most cancer sites, which notably precluded separate analyses in men and women. Small numbers also prevented the analysis of rare cancer sites, although some of them were of special interest, such as subtypes of hematological malignancies. These shortcomings will be overcome when a longer follow-up period will become available. In addition, although we used the smallest available census unit, some of the IRIS in Guadeloupe are relatively large, and then probably heterogeneous. Despite these limitations, our study provided for the first time an extensive description of geographical disparities in cancer incidence, in a region where socioeconomic and environmental issues are major concerns. Although the identification of explanatory factors was out of the scope of the present study, we nevertheless highlighted areas of special interest and put forward some hypotheses that warrant to be further investigated in more in-depth analyses. Etiological studies are planned for some cancer sites. Furthermore, a mapping of the use of pesticides, which take into account the diversity of pollutants and their historical use is in progress, and will allow ecological studies. Taking together, these findings will inform health policies in the territory.

## Supplementary Information

**Additional file 1.**

**Additional file 2.**

## Data Availability

The datasets analyzed for the current study are available from the corresponding author on reasonable request

## Abbreviations

AHC: Ascendant Hierarchical Clustering
BYM: Besag York and Mollié
ICD-O3: International Classification of Diseases for Oncology 3
IRIS: Ilots regroupés pour l’information statistique
SIR: Standardized Incidence Ratio
