# Supplementary material for: Geographical variations of cancer incidence in Guadeloupe, French West Indies
Source: BMC Cancer. 2022 Jul 18;22:783. doi: 10.1186/s12885-022-09886-6 (PMC9290250; doi:10.1186/s12885-022-09886-6)
Supplement: Supplementary file 1 — Additional file 1. [file 12885_2022_9886_MOESM1_ESM.pdf]

Table S1: Sociodemographic characteristics of the 6 clusters

	Cluster 1	Cluster 2	Cluster 3	Cluster 4	Cluster 5	Cluster 6
Population	29157	203578	59513	95890	11173	2808
Women (%)	52.65%	53.45%	55.91%	53.69%	52.28%	48.07%
Population aged 19 or less (%)	28.39%	28.24%	28.70%	27.32%	25.21%	17.72%
Population aged 20 to 64 (%)	55.61%	57.44%	56.03%	56.48%	53.31%	58.48%
Population aged 65 and over (%)	16.01%	14.31%	15.27%	16.20%	21.48%	23.80%
Unemployed (%)	23.63%	18.36%	23.40%	21.64%	18.92%	15.18%
Lower secondary education or less (%)	52.99%	43.60%	47.54%	48.02%	60.24%	61.36%
Working population over 15-year-old						
Farmers and fishermen (%)	1.83%	1.47%	1.97%	1.76%	9.15%	7.14%
Craftsmen, salesmen and managers (%)	10.32%	9.82%	10.23%	8.24%	11.12%	13.94%
Professionals (%)	5.96%	9.64%	6.83%	8.65%	5.20%	4.83%
Technicians and associate professionals (%)	18.30%	23.94%	22.10%	22.68%	14.74%	9.12%
Clerks, service and sale workers (%)	39.56%	35.02%	36.82%	37.20%	36.35%	39.97%
Blue-collar workers (%)	24.04%	20.11%	22.06%	21.46%	23.44%	25.00%
