# Supplementary figures and images for: Geographical variations of cancer incidence in Guadeloupe, French West Indies
Source: BMC Cancer. 2022 Jul 18;22:783. doi: 10.1186/s12885-022-09886-6 (PMC9290250; doi:10.1186/s12885-022-09886-6)

Figure S1: Map of the archipelago of Guadeloupe, F.W.I


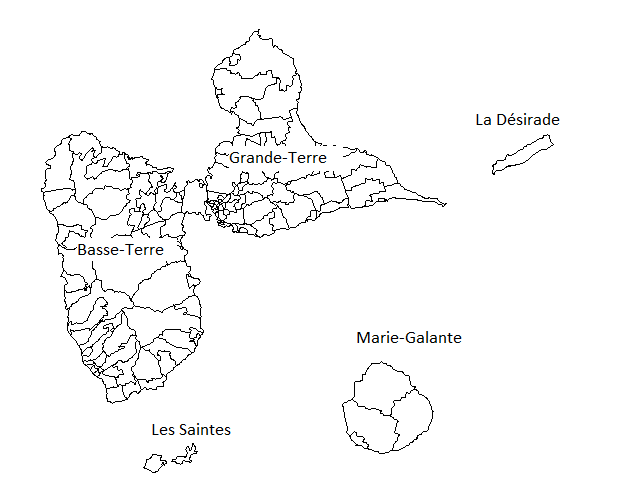

Supplement: Supplementary file 2 — Additional file 2. [file 12885_2022_9886_MOESM2_ESM.docx]
